# Bursting Regimes in a Reaction-Diffusion System with Action Potential-Dependent Equilibrium

**DOI:** 10.1371/journal.pone.0122401

**Published:** 2015-03-30

**Authors:** Stephen R. Meier, Jarrett L. Lancaster, Joseph M. Starobin

**Affiliations:** Department of Nanoscience, Joint School of Nanoscience and Nanoengineering, The University of North Carolina at Greensboro, Greensboro, NC, USA; Georgia State University, UNITED STATES

## Abstract

The equilibrium Nernst potential plays a critical role in neural cell dynamics. A common approximation used in studying electrical dynamics of excitable cells is that the ionic concentrations inside and outside the cell membranes act as charge reservoirs and remain effectively constant during excitation events. Research into brain electrical activity suggests that relaxing this assumption may provide a better understanding of normal and pathophysiological functioning of the brain. In this paper we explore time-dependent ionic concentrations by allowing the ion-specific Nernst potentials to vary with developing transmembrane potential. As a specific implementation, we incorporate the potential-dependent Nernst shift into a one-dimensional Morris-Lecar reaction-diffusion model. Our main findings result from a region in parameter space where self-sustaining oscillations occur without external forcing. Studying the system close to the bifurcation boundary, we explore the vulnerability of the system with respect to external stimulations which disrupt these oscillations and send the system to a stable equilibrium. We also present results for an extended, one-dimensional cable of excitable tissue tuned to this parameter regime and stimulated, giving rise to complex spatiotemporal pattern formation. Potential applications to the emergence of neuronal bursting in similar two-variable systems and to pathophysiological seizure-like activity are discussed.

## Introduction

Understanding neural activity is an endeavor spanning several decades of research. Promising advances have been made in modeling both individual neurons as well as the combination of neurons making up a network. The goal of such work is to understand how our brains store and access information through identifying the internal and external factors which play important roles in these processes. In particular, synchronization of the electrical disturbances in neurons, or action potentials, is believed to play a crucial role in memory formation [1].

By introducing additional nonlinearities to the governing equations, a strong tendency toward synchronization in one-dimensional cables was previously observed within the “soliton-like regime” of the Fitzhugh-Nagumo model [2]. Such examples of synchronization within simple two-variable systems are of great interest for studying memory formation, and in this paper we present a physiologically-motivated modification to a similar two-variable system: the Morris-Lecar (ML) model [3, 4]. The ML model represents a reduced system based on the Hodgkin-Huxley model [5], empirically obtained to describe the voltage dynamics in the squid giant axon.

Recently, Gonzalez-Perez and collaborators [6] presented an experimental study on the behavior of action potential propagation within invertebrates in which it was shown that contrary to some well-known predictions of the Hodgkin-Huxley model, it is possible for action potential pulses to “pass through” each other instead of annihilating upon contact. This soliton-like behavior motivated the authors to abandon a description of their results in terms of Hodgkin-Huxley-like models and employ a model for action potentials in terms of soliton-like sound pulses [6]. The authors did concede, however, that certain results [2] suggest Hodgkin-Huxley-like models *could* support such soliton-like behavior with suitable modifications to parameters. The present work serves as a bridge between recent experimental studies [6] and well-known theoretical results involving these soliton-like regimes [2, 7] by demonstrating how the soliton-like regime emerges within the Morris-Lecar model when leading-order effects due to small neurons are included.

While the Morris-Lecar model is used as a particular example, the main goal of this paper is to explore a technique for incorporating voltage-dependent Nernst potentials into Hodgkin-Huxley-like models. The observation that a variable Nernst potential affects ionic relaxation times has been stated previously by Cressman *et al.* [8, 9]. Due to the very small size of brain axons, the cellular Nernst equilibrium potential is expected to change in response to considerable intracellular charge depletion [10]. Here, we wish to account for this effect in neurons with radii several orders of magnitude smaller than that of the squid giant axon. Within this regime, intracellular charge depletion becomes significant. Accounting for intracellular charge depletion also helps to quantify variations in cell size due to cell swelling which has been observed during epileptic seizures [8, 9].

The paper is organized as follows: The Analysis section provides context for our work, containing a brief overview of Hodgkin-Huxley-like, reaction-diffusion models, as well as a discussion of neuronal bursting and how our approach enables us to investigate this phenomenon. A scheme for incorporating a voltage-dependent Nernst potential into models derivable from the Hodgkin-Huxley system is proposed, and an explicit implementation in the Morris-Lecar model is presented with a discussion of some of the immediate consequences of this generalization. Detailed results from these investigations are reported. Finally, our conclusions are contained in the Discussion.

## Analysis

Memory formation [11] and memory retention have been linked to neural synchronization since the introduction of the “binding problem,” [12] which concerns how the brain constructs effective equivalence classes of objects deemed “similar.” Support for the link between memory and neural synchronization has strengthened over the years, but the exact spatiotemporal dynamics and phase-locking characteristics have yet to be realized are expected to be extremely complex. The system is quite delicate, as deviations from physiologically acceptable conditions can result in memory distortion or impairment [13]. Understanding how these networks can erode in time will help in developing proactive measures to prevent irreversible network damage.

When building successful models, it is imperative to understand the fundamental constituents in great detail. Regarding electrical activity in the brain, the basic building blocks are excitable cells. Hodgkin and Huxley [5] pioneered significant progress in this realm with the introduction of a semi-empirical set of differential equations describing the voltage dynamics in a squid giant axon. The essential ingredients for generating an action potential in a single, excitable cell are a fast inward ionic current followed by a slower outward ionic current. Additional physical currents may also exist, but the key feature is a “lumping” of the dominant currents into “fast” and “slow” groups. This qualitative grouping of many different physical processes into two groups is the basis for many of the two-variable systems which were later introduced as qualitatively similar models [3, 14–16] whose technical simplicity allowed for sophisticated mathematical analysis.

There are many different techniques for extracting a short list of rules from the differential equations governing single neurons which can be applied to a neural network representation such as a cellular automaton [17] or mean-field model [18]. In retaining the full range of single-cell dynamics one must choose between a discrete network representation or a continuous network representation via reaction-diffusion theory. Reaction-diffusion systems provide a universal network structure upon which one may unambiguously investigate various neural coupling strengths via different diffusion profiles. Discrete networks provide a much wider array of possible network structures, and this may naturally result in structurally-dependent coupling profiles. However, it should be noted that, when discretized for numerical simulation, continuous reaction-diffusion systems are nothing more than special cases of discrete networks, and the distinction between the two becomes less apparent. In the present work, our focus will be in exploring the predictions of the continuum limit through numerical work involving a discrete representation of a continuous system.

Network instabilities and abnormalities are thought to be critical features in any detailed explanation of mental diseases such as epilepsy and Alzheimer’s disease. Research into seizure activity [19] suggests that the slight variations of cellular resting potentials due to changes in ionic concentrations during excitation events have observable consequences which are not predicted by conventional models employing static Nernst potentials.

### Charge depletion

During the process of an action potential, charges move across the cell membrane through ionic channels. If the fraction of total charge leaving the cell is substantial, the cell becomes significantly charge-depleted. For large-diameter axons this effect is minimal. However, for smaller neurons such as those in the neocortex, this effect may not be negligible. A substantial charge depletion would dramatically affect the resting potential of the ionic channels [10]. We quantify charge depletion *δ* as the ratio of surface charge to the amount of internal charge at some constant voltage *V*. It is useful to define,

$$\begin{array}{ccc} Q_{s} & = & VC(\pi dL), \end{array}$$ (1)

$$\begin{array}{ccc} Q_{i} & = & F\left[M\right]\left(\frac{\pi d^{2}L}{4}\right), \end{array}$$ (2)

$$\begin{array}{ccc} \delta & = & Q_{s}/Q_{i}=\beta \left(1/d\right), \end{array}$$ (3)

where *Q*_*s*_ is the amount of charge stored on the cell surface for a capacitance (per unit area) *C*, *Q*_*i*_ is the amount of intracellular charge found for an ionic concentration density [*M*], *F* is Faraday’s constant, *d* is the cell’s diameter, *L* is the length of the cell, and *β* = (4*V C*)/(*F*[*M*]). For simplicity, we consider a cylindrical axon.

The diameters of typical axons in the neocortex are roughly three orders of magnitude smaller than the diameter of the squid giant axon [20, 21]. Therefore, *δ* is not small and charge depletion cannot be neglected. To take this into consideration we propose a method for introducing dynamical shifts in Nernst potential which are functions of the instantaneous trans-membrane potential. While the cell membrane may contain many different ionic channels, we model the shift in the effective Nernst potential of the entire membrane.

### Nernst potential shift

Conventional modeling of neuronal excitation takes place on many different levels of detail [22], from the physiologically-detailed and mathematically cumbersome models [23–25] to the qualitatively accurate but mathematically transparent systems [14–16, 22]. A popular class of models is based on the representation of the excitable cell as a circuit in which separate channels exist for each important group of charge-carrying ions. A few well-known examples are the Hodgkin-Huxley [5] and Morris-Lecar [3] models. The fundamental equation of any model representable as a circuit is conservation of charge, which may be written as a differential equation for membrane potential *V* as [20],

$$\begin{array}{c} C\dot{V}=-G_{\text{eff}}\left(V-V_{\text{eq}}\right), \end{array}$$ (4)

where the effective conductance *G*_eff_ and the equilibrium membrane potential *V*_eq_ are given by

$$\begin{array}{ccc} G_{\text{eff}} & = & \sum_{i=1}^{n}G_{i}, \end{array}$$ (5)

$$\begin{array}{ccc} V_{\text{eq}} & = & \frac{1}{G_{\text{eff}}}\sum_{i=1}^{n}G_{i}V_{i}, \end{array}$$ (6)

with *G*_*i*_ being the channel conductance of the *i* ^th^ ionic channel, *n* being the total number of ionic channels embedded within the cell membrane, and *V*_*i*_ being the Nernst potential for the *i* ^th^ ionic channel.

For passive channels *G*_*i*_ is a constant value, and the channel acts like a simple, Ohmic resistor. When the *i* ^th^ ionic channel is active, *G*_*i*_ becomes a function of one or more gating-variables, each of which depends on the membrane potential. The quantity *G*_eff_ is the total conductance over all ionic channels, and *V*_eq_ is the average Nernst potential (weighted by channel conductance). In order for the Nernst potential of a particular ionic channel to remain constant, the intracellular and extracellular ion concentrations must not change by a significant amount over the course of an action potential (*δ* ≪ 1). On the contrary, for substantial charge depletion, *δ* becomes on the order of unity.

### Conventional Morris-Lecar model

In this paper, we will incorporate effects due to significant charge depletion into the two-variable Morris-Lecar model [3, 4],

$$\begin{array}{ccc} C\dot{V} & = & \left[-G_{\text{eff}}\left(V,W\right)\left(V-V_{\text{eq}}\left(V,W\right)\right)\right]+I_{\text{app}}\left(t\right), \end{array}$$ (7)

$$\begin{array}{ccc} \dot{W} & = & \left(W_{\infty }\left(V\right)-W\right)/\tau_{W}\left(V\right), \end{array}$$ (8)

originally obtained from the study of barnacle muscle fibers. Here, *V* is the membrane potential, *W* is a dimensionless gating variable corresponding to the inhibitory response of the potassium channel, and *I*_app_(*t*) is an applied, stimulation current. In this two-variable system, the calcium dynamics is assumed to act on such short timescales that the calcium channel instantaneously finds its voltage-dependent equilibrium state, so *M*_∞_(*V*) has no intrinsic dynamics. The potassium dynamics is modeled through the evolution of the dynamical gating variable *W*. The explicit forms for the effective conductance, equilibrium potential and other voltage-dependent functions are

$$\begin{array}{ccc} G_{\text{eff}}\left(V,W\right) & = & g_{Ca}M_{\infty }\left(V\right)+g_{K}W+g_{L},, \end{array}$$ (9)

$$\begin{array}{ccc} V_{\text{eq}}\left(V,W\right) & = & \left(g_{Ca}M_{\infty }\left(V\right)V_{Ca}+g_{K}WV_{K}+g_{L}V_{L}\right)/G_{\text{eff}}, \end{array}$$ (10)

$$\begin{array}{ccc} M_{\infty }\left(V\right) & = & \frac{1}{2}\left(1+\tanh\left[\frac{V-V_{1}}{V_{2}}\right]\right), \end{array}$$ (11)

$$\begin{array}{ccc} W_{\infty }\left(V\right) & = & \frac{1}{2}\left(1+\tanh\left[\frac{V-V_{3}}{V_{4}}\right]\right), \end{array}$$ (12)

$$\begin{array}{ccc} \tau_{W}\left(V\right) & = & \frac{1}{\varphi }\text{sech}\left(\frac{V-V_{3}}{2V_{4}}\right). \end{array}$$ (13)

**Fig 1:**
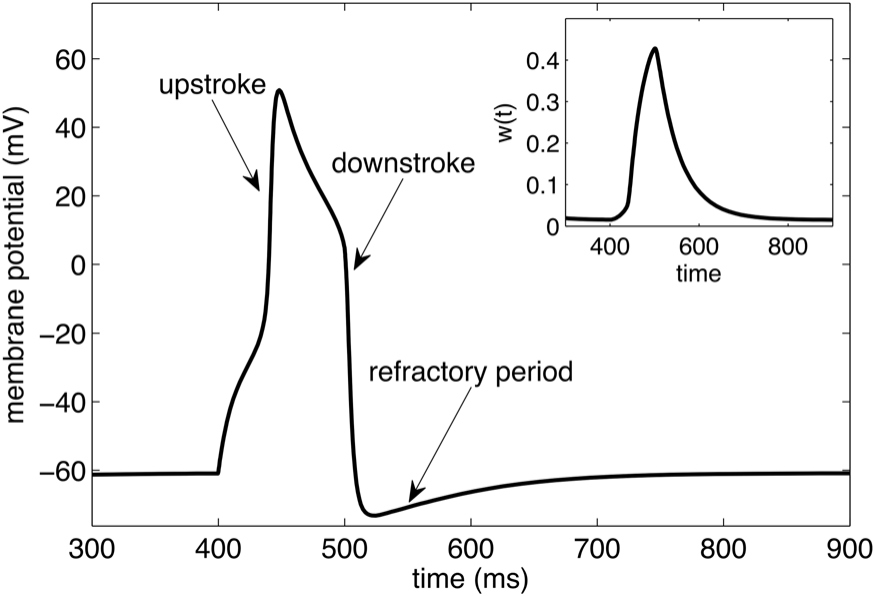
Action potential within Morris-Lecar model. A stimulus is applied to the resting cell at *t* = 400ms. Corresponding behavior of recovery variable *W*(*t*) is shown in the inset.

Typical values for the parameters are shown in Table 1. Using these dimensions, voltage is measured in mV and time in ms. A typical action potential due to a short stimulation current is shown in Fig. 1.

**Table 1:** Typical values for the Morris-Lecar model, Equations (7)–(8).

Parameter	Value
*C*	20 *μ*F/cm^2^
*ϕ*	0.04
*g*_*Ca*_	4.4 *μ*S/cm^2^
*g*_*K*_	8 *μ*S/cm^2^
*g*_*L*_	2 *μ*S/cm^2^
*V*_*Ca*_	130 mV
*V*_*K*_	-84 mV
*V*_*L*_	-60 mV
*V*_1_	-1.2 mV
*V*_2_	18 mV
*V*_3_	2 mV
*V*_4_	30 mV

### Bursting and synchronization in discrete and continuous networks

The term “bursting” typically refers to rapid voltage oscillations which are modulated by lower-frequency oscillations. Of particular relevance to the present work, it was found recently [26] that bursting may emerge in single-cell models when the extracellular ionic concentrations are allowed to vary. Essentially, this variation in ionic concentrations gives rise to the low-frequency oscillations which modulate the bursting. It should be noted that this variation in ionic concentrations gives rise to a varying Nernst potential. Our approach can be viewed as an effective description in which the degrees of freedom corresponding to ionic concentrations have been integrated out of the equations of ionic motion.

Extended systems of coupled neurons, or neural networks, allow for a transmembrane potential difference to travel across multiple neurons by means of a propagating excitation wave. This process may result in synchronized electrical activity for groups of neighboring neurons. Such a synchronized excitation of neighboring cells is believed to play a central role in neural communication [27].

Bursting has previously been observed in discrete networks of mutually inhibitory oscillators [28] and is responsible for pattern generation as seen within many different biological neural networks [29]. To investigate bursting, Skinner *et al.* used a Morris-Lecar-based network and explored inhibition through synaptic coupling by introducing the following source

$$\begin{array}{c} I_{\text{syn}}=m\left(\hat{V}\right)\left(V-V_{\text{syn}}\right), \end{array}$$ (14)

where *I*_syn_ is the synaptic current provided by the inhibitory neuron, $m(\hat{V})$ is a step-wise function which is zero below a particular voltage threshold and a positive constant above the threshold, and $\hat{V}$ is the voltage of the inhibitory neuron. It was demonstrated that such a coupling allows for frequency control analogous to a neuromodulator where synaptic currents affect the intrinsic properties of single neurons as described in [30].

Unlike the work of Skinner *et al.* we will ultimately discover how bursting regimes for a single cell can emerge through coupling to an extended system in a continuous reaction-diffusion model, where coupling between neuronal cells is introduced by a diffusion term in a nonlinear diffusion equation

$$\begin{array}{c} \frac{\partial V(x,t)}{\partial t}=D\frac{\partial^{2}V\left(x,t\right)}{\partial x^{2}}+f\left(V\right), \end{array}$$ (15)

When a reaction-diffusion system is written in discrete form, suitable for numerical analysis, the effective coupling term (to second order) can be written in the form,

$$\begin{array}{ccc} I_{\text{syn}} & = & D\frac{\partial^{2}V\left(x\right)}{\partial x^{2}}, \end{array}$$ (16)

$$\begin{array}{ccc} & \to & \frac{D}{\Delta x^{2}}\left(V_{i+1}-2V_{i}+V_{i-1}\right), \end{array}$$ (17)

$$\begin{array}{ccc} & = & \frac{-2D}{\Delta x^{2}}\left(V_{i}-\left[V_{i+1}+V_{i-1}\right]/2\right), \end{array}$$ (18)

$$\begin{array}{ccc} & \equiv & \Gamma \left(D\right)\left(V_{i}-V_{\text{avg}}\right), \end{array}$$ (19)

where Γ(*D*) plays a role analogous to the quantity $m(\hat{V})$ in Equation 14. If the model is extended to include nonlinear diffusion, *D* = *D*(*u*), then Γ(*D*) acquires an implicit dependence on membrane potential, though we will not consider nonlinear diffusion in this work. Furthermore, we note that the term “diffusion” in this continuum model is employed in a coarse-grained sense. Within the network language, we are only considering synaptic coupling. From the network point of view, diffusion of ions through extracellular space is seemingly absent. However, at the level of complexity of this model, these effects could be studied by adjusting the form of the effective diffusion profile.

### Morris-Lecar model with adaptive Nernst potential

A variable Nernst potential across one or more ionic channels can be incorporated into Equation (4) by introducing a shift *V*_*δ*_ as

$$\begin{array}{c} C\dot{V}=-G_{\text{eff}}\left(V-\left[V_{\text{eq}}+V_{\delta }\right]\right). \end{array}$$ (20)

The total trans-membrane potential difference from equilibrium, (*V* − *V*_eq_), can be considered as the driving force of this nonlinear system. As the trans-membrane potential difference increases, the amount of charge stored on the cell surface also increases. This causes the concentration of intracellular ions to decrease, resulting in an elevated Nernst potential for positive ions. Using the Nernst equation [10] one finds the leading-order correction to the Nernst potential *V*_*i*_ of a single channel due to significant charge depletion *δ* = Δ*Q*_*i*_/*Q*_*i*_ to be,

$$\begin{array}{c} \Delta V_{i}\approx -\frac{RT}{z_{i}F}\left(\frac{\Delta Q_{i}}{Q_{i}}\right), \end{array}$$ (21)

where Δ*V*_*i*_ is the Nernst shift for the *i*th ionic channel, *R* is the ideal gas constant, *z*_*i*_ is the charge value for the *i*th ionic channel, *T* is temperature (assumed constant), and *F* is Faraday’s constant. Any flux of charge leaving or entering through the cell membrane is a result of the total potential difference across the membrane surface,

$$\begin{array}{c} \Delta Q_{i}\propto \left(V-V_{\text{eq}}\right). \end{array}$$ (22)

Thus, for each ionic channel, the Nernst shift will be proportional to the total trans-membrane potential difference. Because this is true for any ionic channel with variable Nernst potential, the effective Nernst shift for the system as a whole, *V*_*δ*_, will also depend on the total trans-membrane potential,

$$\begin{array}{c} V_{\delta }=\alpha \left(V_{0}-V\right), \end{array}$$ (23)

where *α* and *V*_0_ are constant parameters. Letting *ζ* represent the ratio of the average Nernst shift to the total trans-membrane potential,

$$\begin{array}{c} \zeta =\frac{V_{\delta }}{\left(V-V_{\text{eq}}\right)}, \end{array}$$ (24)

we stress that the form for *ζ* is a consequence of the general considerations leading to Equation (21). Our description consists of an effective feedback loop whereby variation in the Nernst potentials for one or more cells is represented as a closed function of the instantaneous transmembrane potential. This minimally complex representation of *ζ* in Equation (24) allows us to probe the qualitative effects that arise when considering small cells without restricting attention to one type of ionic channel or a particular class of neurons. Interesting studies [26] have obtained bursting in single cells by introducing dynamics to several ion concentrations in a Hodgkin-Huxley model. Here we will examine the effects of significant charge depletion in small neurons. Furthermore, it should be noted that within our continuum description, a single fundamental “cell” corresponds to many individual neurons. Thus, while bursting of a single neuron is physiological, bursting of a fundamental cell consisting of thousands of neurons is potentially pathological.

Conservation of charge in a circuit-based model with variable Nernst potential in the form described by Equation (20) and Equation(23) can thus be written as,

$$\begin{array}{c} C\dot{V}=\left(1-\zeta \right)\left[-G_{\text{eff}}\left(V-V_{\text{eq}}\right)\right]. \end{array}$$ (25)

A similar type of modification has been studied in the Fitzhugh-Nagumo model, where a “soliton-like regime” was discovered [7]. This soliton-like regime represents a region in parameter space of a modified Fitzhugh-Nagumo model in which waves appear to reflect from no-flux boundary conditions. This is to be distinguished from the “soliton model” which is discussed in Ref. [6], which is based on different physical principles. The connection of the present work to the soliton-like regime in the Fitzhugh-Nagumo model is explored in the next section. For single-cell and network simulations in this paper, Equation (25) is applied to the two-variable Morris-Lecar model [3]. When modified using Equation (25), the Morris-Lecar system given by Equations (7)–(8) becomes,

$$\begin{array}{ccc} C\dot{V} & = & \left(1-\zeta \right)[-G_{\text{eff}}\left(V-V_{\text{eq}}\right)], \end{array}$$ (26)

$$\begin{array}{ccc} \dot{W} & = & \left(W_{\infty }-W\right)/\tau_{W}, \end{array}$$ (27)

with *V*_*δ*_ given by

$$\begin{array}{ccc} V_{\delta } & = & \alpha (V_{0}-V), \end{array}$$ (28)

with Equations (9)–(13) unchanged. Note that rather than being specific to the Morris-Lecar model, Equation (25) represents a general framework for incorporating a voltage-dependent Nernst equilibrium into any conductance-based, or Hodgkin-Huxley-like, model. We have chosen to explore these effects using the Morris-Lecar model for its convenient balance between mathematical simplicity and biological relevance. While not as detailed as the Hodgkin-Huxley system, its parameters are based on biological quantities and not commonly viewed as arbitrarily tunable parameters. However, as a two-variable system the Morris-Lecar model shares a qualitative simplicity with other mathematically idealized models such as the Fitzhugh-Nagumo system.

### Derivation of “Soliton-like regime” in Fitzhugh-Nagumo model

Mornev and collaborators [31] considered a modified form of the Fitzhugh-Nagumo equations,

$$\begin{array}{ccc} \frac{du}{dt} & = & f\left(u\right)-v, \end{array}$$ (29)

$$\begin{array}{ccc} \frac{dv}{dt} & = & \epsilon \left(u\right)\left[\zeta u-v\right], \end{array}$$ (30)

where *f* (*u*) = (*u* − *m*_0_) (*u* − *m*_1_) (*u* − *m*_2_), and *ϵ* (*u*) ≡ *ϵ*_0_ *g*(*u*), with

$$\begin{array}{c} g\left(u\right)=\left(1+\lambda \left[2-\tanh\left[\frac{u+0.04}{0.01}\right]+\tanh\left[\frac{u-0.75}{0.1}\right]\right]\right), \end{array}$$ (31)

for some constants *ϵ*_0_ and *λ*. Rescaling the time variable according to

$$\begin{array}{c} d\tau =g\left(u(t)\right)dt, \end{array}$$ (32)

Equations (30)–(31) become

$$\begin{array}{ccc} \frac{du}{d\tau } & = & \frac{1}{g(u)}\left[f(u)-v\right], \end{array}$$ (33)

$$\begin{array}{ccc} \frac{dv}{d\tau } & = & \epsilon_{0}\left[\zeta u-v\right], \end{array}$$ (34)

so that with rescaled time, the *u*-dependence introduced to *ϵ*, responsible for the behavior characterstic of the “soliton regime” examined in [31], can be recast in the form of an effective shift in Nernst potential by the identification

$$\begin{array}{c} \frac{1}{g(u)}\to 1-\zeta , \end{array}$$ (35)

where in the *ζ* is a ratio of two linear functions of *u*, according to Equations (23)–(24), and *u* plays the role of *V* in the Fitzhugh-Nagumo system. Performing a Taylor expansion of the hyperbolic functions around *u* ≈ −0.04 or *u* ≈ 0.75 reduces *g* ^−1^(*u*) to the same functional form as 1 − *ζ*, indicating that the “soliton-like” effects previously observed can be understood from a physiological perspective as a result of an adaptive Nernst potential which becomes more pronounced in its effects for smaller neurons. We strongly emphasize that “soliton-like behavior” is a term used by Mornev *et al.* to describe a dynamical regime within continuous reaction-diffusion systems and is entirely distinct from the so-called “soliton model” used in Ref. [6], which is a model for neural signals based on entirely different physical principles. Systematically incorporating further realistic complications such as nonlinear diffusion to our model is a direction for future research.

## Results

### Single-cell dynamics

In this section, we wish to explore some basic properties of the Morris-Lecar system with the addition of an adaptive Nernst equilibrium by examining the equations governing a single, excitable cell. While the context of the present work lies in studying behavior of neurons in the brain, we shall employ the standard Morris-Lecar parameters as a way to demonstrate the substantial effects caused by the introduction of a variable Nernst potential while minimizing the number of free parameters. The implications of our results outside of the usual domain of relevance for the Morris-Lecar model are explored in the Discussion, but we note here that the present goal is a demonstration of the wide variety of interesting behaviors that can be captured with a continuous reaction-diffusion system which has been suitably modified to incorporate the dominant physical effects due to smaller size of neurons.

As a starting point, we refer the reader to Fig. 1, which shows the generic behavior observed in the conventional Morris-Lecar system. An external stimulation current can be applied to raise the membrane potential *V* above the model’s threshold at which a rapid rise in potential (the “upstroke”) occurs, causing an increase in the potassium current. This rising potassium current brings the membrane potential back down (the “downstroke”), overshooting equilibrium and resulting in a recovery time (“refractory period”) during which no further stimulation generates an action potential. This basic picture of an action potential event is characteristic of virtually all common models used to study electrical activity in cardiac and muscular cells, and we now wish to explore how this picture changes when the Nernst potential is allowed to vary due to the charge depletion expected to occur within smaller neurons.

**Fig 2:**
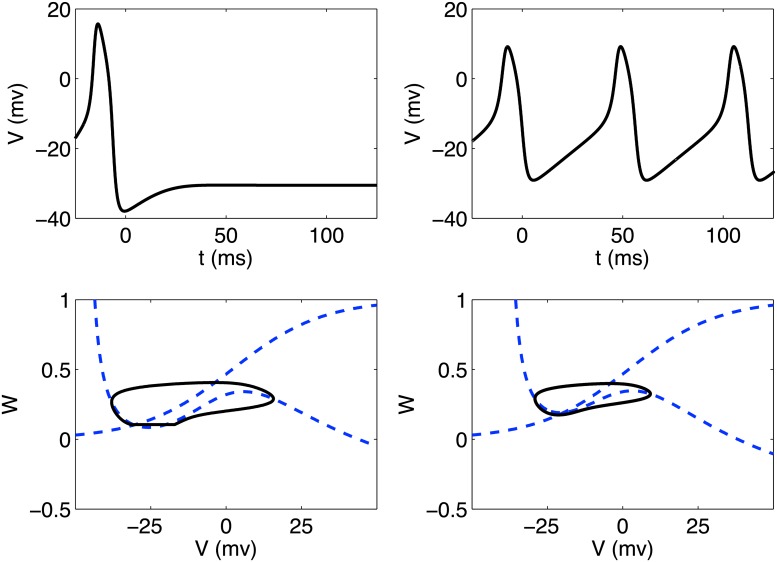
Time series and phase space diagrams for modified Morris-Lecar model. Upper left: Single neuron (*α* = 0.7, *V*_0_ = 6.2mV) firing once after stimulus is applied at *t* = 0ms and approaching a stable equilibrium. Upper right: Single neuron (*α* = 1 and *V*_0_ = 6.2mV) entering a stable limit cycle after initial stimulus. Phase space trajectories for each case are shown in the panel below the corresponding time-series plot.

Our modified system contains two free parameters, namely *α* and *V*_0_. Solving Equations (26)–(27) at different points in (*V*_0_, *α*) space reveals two qualitatively different regimes of behavior. A region exists where most initial conditions fall into a stable limit cycle without any external stimulation current. In this regime, the system exhibits “autogeneration” of excitations. Outside of this region, the system behaves qualitatively similarly to the standard Morris Lecar model given in Equations (7)–(8). An example of the time-series *V*(*t*) and phase space (*V*, *W*) is shown for each of these regimes in Fig. 2.

**Fig 3:**
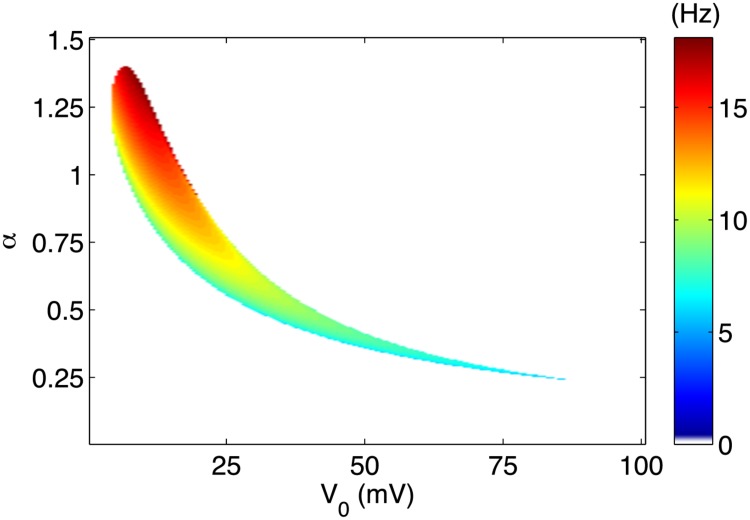
Map in (*V*_0_, *α*) space of resonant frequencies using typical parameters for Morris Lecar model. Regions colored in white correspond to points where no stable limit cycles exist in the absence of a stimulation current.

While a complete characterization of this modified system is beyond the scope of the present work, we sketch a global aspect of the qualitative behavior in Fig. 3 where the natural resonant frequency is shown as a function of position in (*V*_0_, *α*) space. The actual resonant frequencies depicted depend directly on the particular choices of parameters in Table 1, but the for arbitrary choices of parameters, one may expect at least the order of magnitude in variation of resonant frequencies as both *α* and *V*_0_ are varied the range shown in Fig. 3.

**Fig 4:**
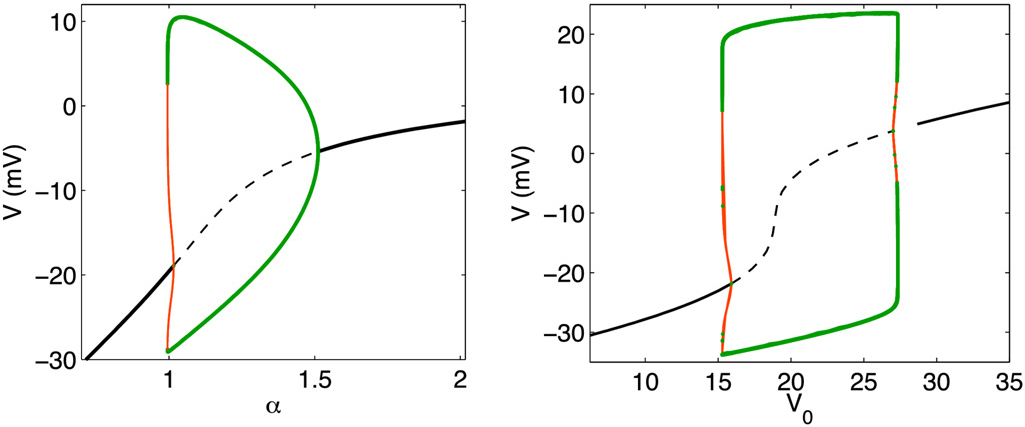
Bifurcation diagrams in extended parameter space. These diagrams, created using Xppaut, depict the occurrence of stable, equilibrium points (solid black line), unstable equilibrium points (dashed black line), stable limit cycle (thick green line) and unstable limit cycle (thin, red line) as *α* (left) and *V*_0_ (right) are varied. From left to right in the left panel, a stable equilibrium branches into an unstable limit cycle through a subcritical Hopf bifurcation point near *α* ≈ 1. As *α* is increased, a stable limit cycle emerges which collapses into a stable equilibrium at the supercritical Hopf bifurcation point near *α* ≈ 1.5.

The auto-generation of excitation pulses produced by Equations (26)–(27) is not entirely different from the behavior produced by the standard Morris Lecar model (c.f., Equations (7)–(8)) in the presence of a constant stimulation current. Indeed, a bifurcation diagram with respect to either *α* or *V*_0_ demonstrates the emergence of a stable limit cycle within a range of values. Fig. 4 depicts these bifurcation diagrams which may be compared to the standard Andronov-Hopf bifurcation observed within the conventional Morris-Lecar model with respect to a varying stimulation current. While mathematically similar, a distinguishing feature of the particular model presented here is that this regime of auto-generation emerges naturally within the extended parameter space of a model which includes the physical effects due to smaller size of neurons. Specifically, these oscillations are driven by a Nernst potential which adapts to the instantaneous charge depletion experienced by the cell during an excitation pulse rather than an external current. Despite this difference in underlying mechanisms, the subcritical Hopf bifurcation that occurs as either *α* or *V*_0_ is varied shows no identifiable differences from the standard bifurcation one finds as an external stimulation current is varied.

The relevance of these findings to actual experimental systems warrants some discussion. A small window of elevated extracellular potassium concentration has previously been discovered through numerical experiments with the Hodgkin-Huxley model [2] in which action potential pulses in a one-dimensional cable reflect rather than annihilate upon collision. This soliton-like behavior is qualitatively similar to the dynamics produced by this model when an extended, one-dimensional cable is considered (see next section). However, we emphasize that the present framework for incorporating an adaptable (averaged) Nernst potential probes somewhat more universal features than changing the concentration of a particular ionic species. The behavior we find here should be characteristic of small neurons, regardless of the particular details regarding individual ionic concentrations.

**Fig 5:**
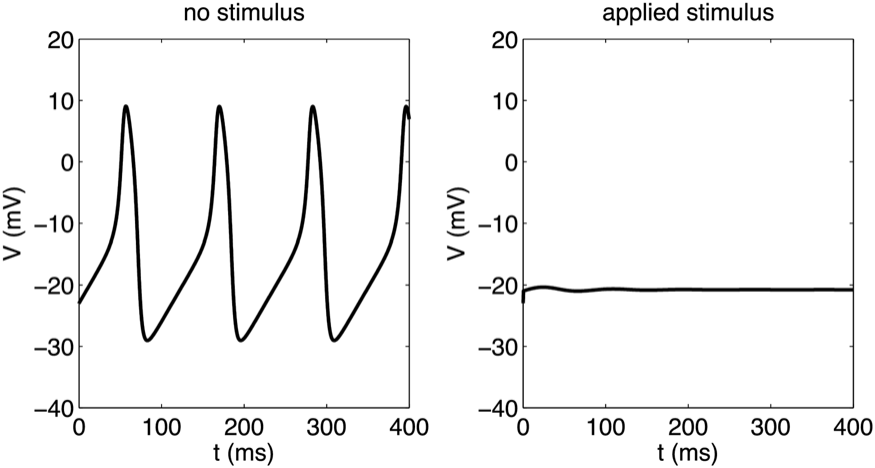
Limit cycle and quiescent state achieved by stimulation. Left: Auto-generation of pulses in modified Morris-Lecar system with *α* = 1, *V*_0_ = 6.2mV. Right: An initial stimulus of 80 *μ*A/cm^2^ applied for 5ms is sufficient to pull the system off the limit cycle to a stable equilibrium.

As a last exploration of the single cell properties, we note that within the limit cycle, a range of stimulation currents may be applied within a particular phase window to send the system to a stable equilibrium point. That is, there is a small window of time during the oscillation for which an applied stimulus can destroy the the sustaining oscillations. Fig. 5 depicts a stable limit cycle for a particular choice of (*V*_0_, *α*) and how this dynamical behavior may be modified when a stimulation current is applied at a certain point in the cycle. Within this “vulnerable” phase window, a sufficient stimulation current can prevent further excitation pulses and cause the membrane potential to asymptotically approach a constant value, as shown in the right panel of Fig. 5. Such a scenario is only expected to occur for (*V*_0_, *α*) chosen close to the boundary depicted in Fig. 3, corresponding to the neighborhood of the subcritical Hopf bifurcation where unstable limit cycles and stable equilibrium points coexist. No similar behavior was observed in the vicinity of the super-critical Hopf bifurcation point (see caption of Fig. 4).

**Fig 6:**
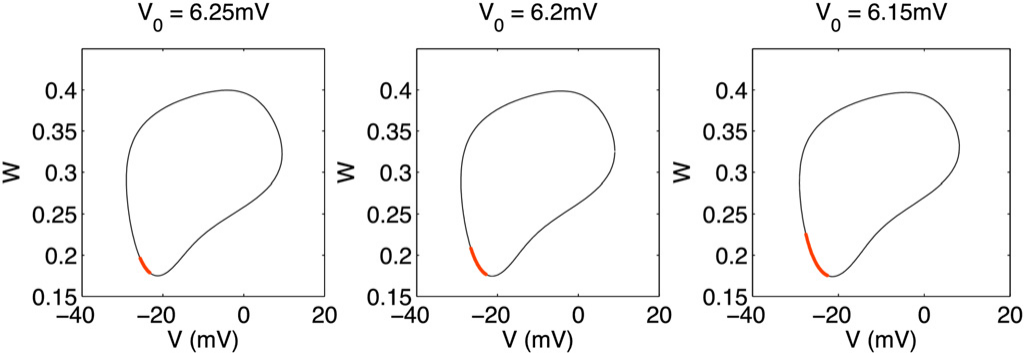
Vulnerable window in the modified Morris Lecar model depicted in phase space. The system’s limit-cycle trajectory for *α* = 1, *V*_0_ = 6.2mV is shown (thin black line) with the vulnerable region indicated by a thick red line for varying values of *V*_0_ with *α* = 1. Within the vulnerable window, a short stimulation takes the system from its stable limit cycle to a stable equilibrium point in phase space. The size of the vulnerable window increases as (*V*_0_, *α*) approaches the Hopf bifurcation point.

To fix this notion of a vulnerable window, we may repeat the calculation leading to Fig. 5 for a fixed stimulation current protocol while varying the point in phase space at which the stimulation is applied. The range of phase space over which this particular stimulation protocol is effective in stabilizing the system is shown in Fig. 6. This window is shown for several values of *V*_0_ in the vicinity of the Hopf bifurcation while keeping *α* fixed. As should be expected, the size of this vulnerable window depends greatly on the proximity of (*V*_0_, *α*) from the Hopf bifurcation, with an increasing window size as one approaches the bifurcation point. This is consistent with the lack of any vulnerable window for points inside the resonant region, since the oscillations are robust to the perturbation of the stimulation current. Far from the boundary outside the resonant region, the vulnerable window, as defined, encompasses the entire orbit since only stable equilibrium points exist as long-time steady-state solutions. Fig. 6 essentially depicts the crossover between these two extremes.

While we have chosen a particular stimulus protocol and varied phase, the general picture of a “vulnerability window” is quite robust and emerges within a measurable fraction of the phase space as a range of vulnerability with respect to variation in any particular parameter of interest. Notably, this “vulnerable window” has been observed experimentally [32] within a space-clamped squid giant axon due to calculations by Rinzel [33] using the Hodgkin-Huxley model suggesting its existence within a particular parameter regime. In the next section, this notion of vulnerability is extended to the context of a one-dimensional cable of excitable tissue and explored as several size-related parameters are varied.

### One-dimensional excitable cable

In this section, we investigate the consequences of the window of vulnerability, depicted in Fig. 6, when the cell being stabilized is coupled to a chain of excitable cells by a diffusive term. Formally, we are investigating a continuous, one-dimensional piece of spatially extended tissue. After discretization for numerical investigations, this cable takes the form of a one-dimensional chain of coupled nonlinear oscillators. Unless otherwise stated, the cable length is taken to be *N*_*x*_ = 119. However, our results are faithful representations of the continuum limit since a change in *N*_*x*_ can be supplemented by an appropriate rescaling of the spatiotemporal mesh to obtain identical results, as discussed below.

As we shall demonstrate, this notion of “vulnerability” extends naturally to a one-dimensional cable through a range of parameter values which allows a localized stimulation to stabilize an entire, synchronized cable. Regarding the experimental relevance of such a vulnerability window in extended systems, previous research has demonstrated the existence of a soliton-like regime close to a subcritical Hopf bifurcation point. Analysis using the Hodgkin-Huxley model predicts this regime to exist within a 0.1 mM concentration window of extracellular potassium [2]. Below this concentration one generically observes standard action potential propagation. Above this region one observes pulse trains of propagating action potentials, which are likely contributors to seizure-like activity. A shift from the single-fire regime to the soliton-like regime, with pulse trains of action potentials, is typically pathophysiological.

Given the clear role action potentials play in formation of memory [1], we are also interested in the possible patterns one could find within this soliton-like regime, for which small diameter axon activity would likely reside. Using the modified Morris-Lecar model, we present a fundamental, biologically-based mechanism for spatiotemporal pattern generation. For our investigation of extended excitable media, we will consider a continuous cable of excitable tissue governed by,

$$\begin{array}{ccc} C\dot{V} & = & D\frac{\partial^{2}V}{\partial x^{2}}+\left(1-\zeta \right)\left[-G_{\text{eff}}\left(V-V_{\text{eq}}\right)\right]+I_{\text{app}}\left(x,t\right), \end{array}$$ (36)

$$\begin{array}{ccc} \dot{W} & = & \left(W_{\infty }-W\right)/\tau_{W}. \end{array}$$ (37)

Normally, the system of Equations (37, 38) has a simple excitation pulse solution in response to an external stimulus. If *α* and *V*_0_ are selected close to an Andronov-Hopf bifurcation point we obtain one stable equilibrium and two limit-cycles (one stable, one unstable) simultaneously [34] for each cell in the cable, as shown in Fig. 4. A particular consequence of each cell lying close to this bifurcation point is a global vulnerability of the entire cable with respect to localized stimulations. To demonstrate this global vulnerability, we consider a cable of length *L* undergoing synchronized oscillations (i.e., each cell in the cable is oscillating in phase with frequency given by Fig. 3) and apply a short stimulation current near the center of the cable at a particular phase of the oscillation. Henceforth, we fix our initial conditions to be *V* (*t* = 0, *x*) = *V*_0_, *W*(*t* = 0, *x*) = *W*_0_, with

$$\begin{array}{ccc} V_{0} & = & -22.9764\text{mV}, \end{array}$$ (38)

$$\begin{array}{ccc} W_{0} & = & 0.1770. \end{array}$$ (39)

**Fig 7:**
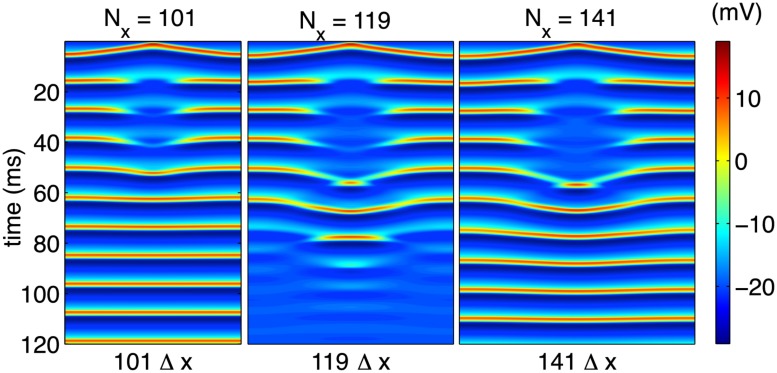
Long-time steady states for cables of varying lengths. Fixing all parameters except cable length generically yields a region of vulnerability in extended cables. For small (left) and large (right) cables, an initial stimulation sufficient to quiesce a single cell causes a transient quiescent region which results in full synchronization. For a range of cable lengths (center) the stimulation results in the entire cable approaching an equilibrium state.

The diffusion constant is fixed to *D* = 0.01cm^2^/s unless otherwise noted, and we take *L* = *N*_*x*_Δ*x* to be the length of the cable, where *N*_*x*_ is the number of spatial points considered. Here the spatial mesh is taken as Δ*x* = 0.1cm, and we employ a time step size Δ*t* = 0.01ms. Fixing *α* = 1 and *V*_0_ = 6.2mV as a representative point in (*α*, *V*_0_) close to the system’s Andronov-Hopf bifurcation point, we proceed to first demonstrate that a fixed stimulation current is able to de-synchronize an entire, extended cable for a fixed range of cable lengths. Fig. 7 schematically depicts this window of vulnerability by applying a stimulation current of amplitude *i*_0_ = 80*μ*A/cm^2^ for a duration *T*_0_ = 1000Δ*t* to the center cell and its three nearest neighbors to the right and left for a total of seven cells. For both sufficiently large and sufficiently small cables, a stimulus able to stabilize a single cell is unable to counteract the cell’s coupling to its oscillating, neighboring tissue (left and right panels of Fig. 7), and the system returns to a synchronized limit cycle. However, for a range of cable lengths, the initial stimulus results in a fully quiescent region which eventually spreads throughout the entire length of the cable (center panel). This resonant effect occurs for a small range in values of *L* given all other parameters fixed.

**Fig 8:**
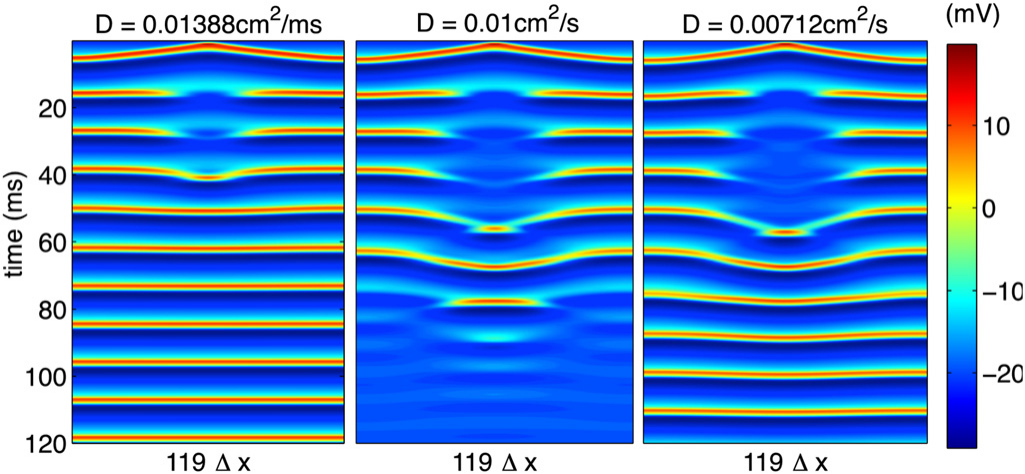
Variation of diffusion constant for fixed cable size. The similarity of these results to those in Fig. 7 shows results consistent with the continuum predictions.

In the continuum limit, with which we are interested, the diffusion constant we have introduced is scalable in the sense that a change in *D* for a system of length *L* = *N*_*x*_Δ*x*,

$$\begin{array}{c} D\to D^{'}, \end{array}$$ (40)

should result in spatiotemporal dynamics equivalent to those in a system of size

$$\begin{array}{c} L\prime =L{\left(\frac{D}{D^{\prime }}\right)}^{1/2}, \end{array}$$ (41)

with diffusion constant *D*. To demonstrate that we are considering discretized systems that effectively represent the continuum limit, we may test this scaling by holding *L* = *N*_*x*_Δ*x* fixed and varying *D* in a manner that should reproduce results equivalent to those depicted in Fig. 7. The results of this variation in diffusion constant are shown in Fig. 8 and support the claim that, by comparison to Fig. 7, these results genuinely represent an accurate description of the continuum limit.

**Fig 9:**
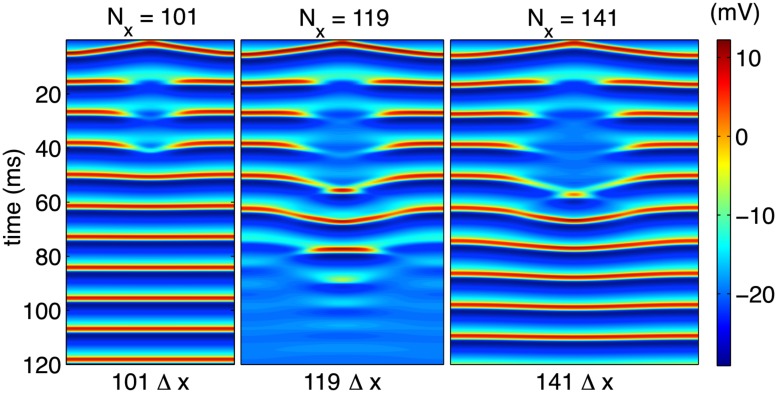
Larger stimulation current (*i*_0_ = 800*μ*A/cm^2^) is used for a shorter time (*T*_0_ = 50Δ*t*) in the middle of the cable. The modified stimulation protocol produces qualitatively similar results to those shown in Fig. 7

While adjusting the diffusion constant does not itself represent a truly independent variation of system properties, we can demonstrate the robustness of our results by modifying the stimulation protocol. The nature of the window of vulnerability is fairly insensitive to changes in the nature of the stimulation, provided the overall charge *Q* = *i*_0_Δ*t* is sufficiently large. Fig. 9 shows a picture qualitatively similar to that in Fig. 7, produced with a larger stimulation current, *i*_0_ = 800*μ*A/cm^2^, applied for a shorter time *T*_0_ = 50Δ*t*.

The general behavior of the model within the regime we have focused is fairly straightforward. As with the single cell, when *α* and *V*_0_ are chosen close to the Hopf bifurcation point (close to the edge of the cloud showing nonzero resonant frequencies in Fig. 3) and a stimulation is applied within the vulnerable phase region (see Fig. 6) in a localized region at the center of an extended, one-dimensional cable, there exists a range of lengths for which the entire cable becomes quiescent due to the stimulation. Outside of this range, for both smaller and larger cable lengths, the synchronized oscillations overtake any transient, locally quiescent behavior. By varying the oscillation phase at which the stimulation is applied and holding all other parameters (cable length, stimulation strength, etc.) fixed, one may construct a global vulnerability picture for the entire cable as was done for the single cell in Fig. 6.

**Fig 10:**
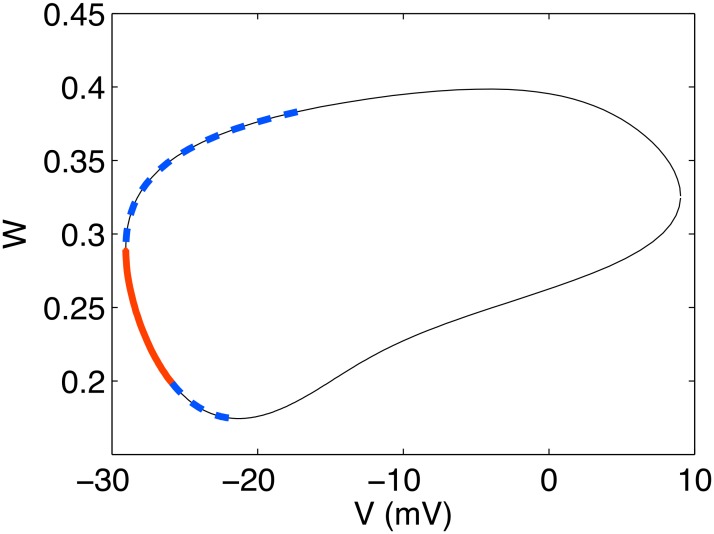
Vulnerable window of the one-dimensional cable shown in phase space. The system’s limit-cycle trajectory for *α* = 1, *V*_0_ = 6.4mV is shown (thin black line) with the vulnerable region indicated by a thick red line. Localized stimulations applied within this window result in a long-time quiescent state for the entire system. Regions of instability are shown in dashed blue line. For all other points (thin black line), localized stimulations only give rise to transient effects, and the entire cable eventually returns to synchronized oscillations.

The global vulnerability picture for *N*_*x*_ = 119, *i*_0_ = 100*μ*A/cm^2^, *T*_0_ = 1000Δ*t* is shown in Fig. 10, and the basic picture is quite similar to that for a single cell. Specifically, a small window of phases exists at which a stimulation may be applied resulting in the entire cable transitioning from synchronized oscillations to a homogeneous, quiescent state. Note that in the case of Fig. 10, this vulnerability refers to a localized stimulation being able to quiesce the entire cable. Far away from this window, the effects due to the localized stimulation are transient, and the entire cable returns to synchronized oscillations at long times. However, a new layer of complication is introduced by the spatial extension of the one-dimensional system. In the region of the transition between these two types of long-time steady states, the system shows extreme sensitivity to the particular details of stimulation. The complex behaviors which can emerge in an extended PDE system have been recently emphasized [35, 36] in numerical studies of the Hodgkin-Huxley system in which the role of noise was investigated when the stimulation current was tuned to be very close to the value for which a subcritical Hopf bifurcation occurs in the system. We emphasize that this proximity to a bifurcation point is likely what gives rise to similarly complex effects in drastically different contexts. Moreover, numerical instabilities arise that make an accurate description of the system at long times practically impossible. The region of phase at which a stimulation gives rise to complex instabilities is shown by the dashed blue line in Fig. 10.

**Fig 11:**
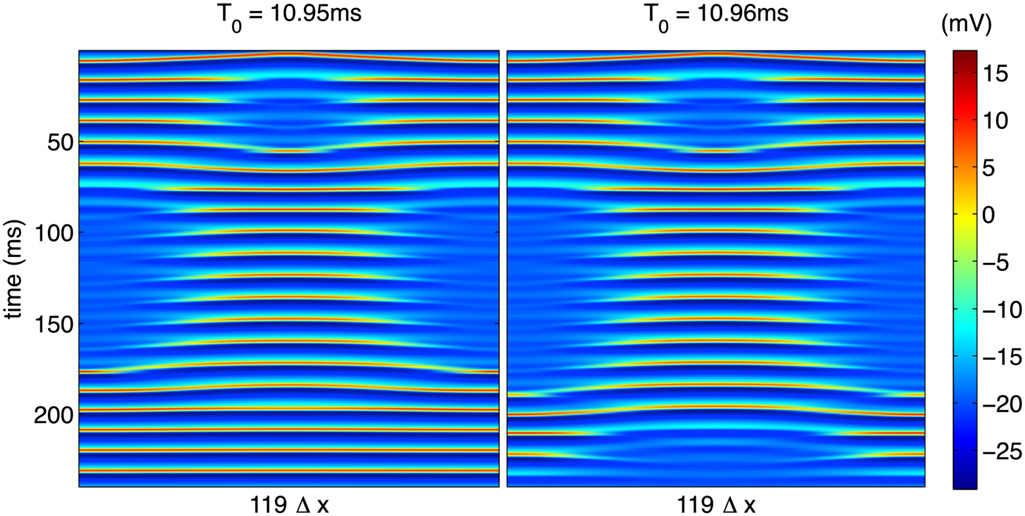
Complex spatiotemporal pattern generated with increased stimulation time near the crossover between quiescent and synchronized steady-states. A standard, second-order stencil was used for evaluation of the spatial derivative.

As an example of the interesting types of behavior contained in this unstable regime, Fig. 11 depicts some extremely long-lived transient behavior. In this case, the cable is extremely sensitive to the time *T*_0_ during which the stimulation is applied with small changes in *T*_0_ corresponding to dramatic changes in the long-time steady state. The persistence of this transient spatiotemporal complexity for long times (tens of oscillations, as shown in Fig. 11) makes an accurate investigation of the dynamics governed by the highly nonlinear partial differential equations, Equations (37)–(38) practically quite difficult, and this sensitivity is discussed in more detail in the following section.

**Fig 12:**
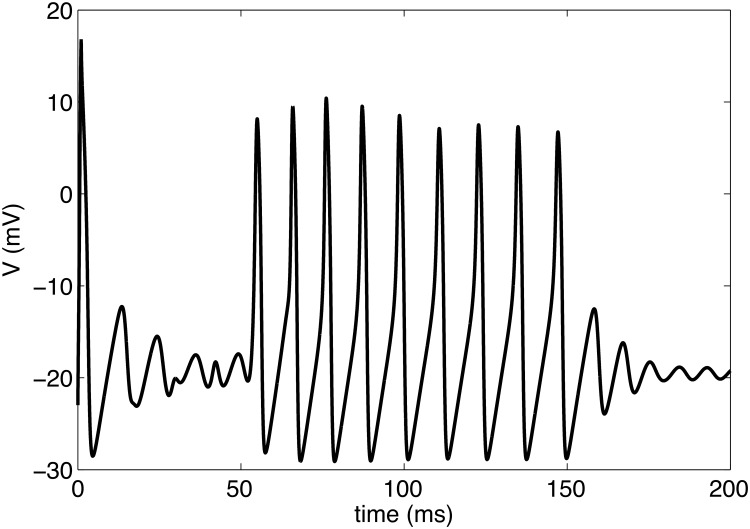
Emergent bursting in the center cell. Time series for the membrane potential *V* at the center cell in the one-dimensional cable shown in Fig. 11.

The possibility of generating such complex spatiotemporal patterns as those shown in Fig. 11 is intriguing in its own right. However, another interesting aspect of this complexity may be seen by considering the time-series for the membrane potential of a single cell. Fig. 12 depicts the membrane potential as a function of time for the center cell in the right-hand panel of Fig. 11. The shape of *V*(*t*) in Fig. 12 is remarkably similar to bursting behavior, which is typically obtained by introducing a third dynamical variable to a two-variable system such as the Morris-Lecar model. Through the propagation of voltage through the extended medium and the complex dynamics generated with the adaptive Nersnt-potential, our model shows the potential to capture bursting. Abstractly, one may think of our (effective) “third equation” as the integrated effects resulting from coupling the center cell to the rest of the cable. Unlike other conventional models for bursting [37, 38], we find bursting to arise in this intrinsically two-variable system through coupling of the cell to an extended system. Interestingly, it has previously been pointed out that within a three-variable model bursting may emerge as a similarly transient phenomenon before the system settles into periodic spiking. [39]

### Numerical Details

**Fig 13:**
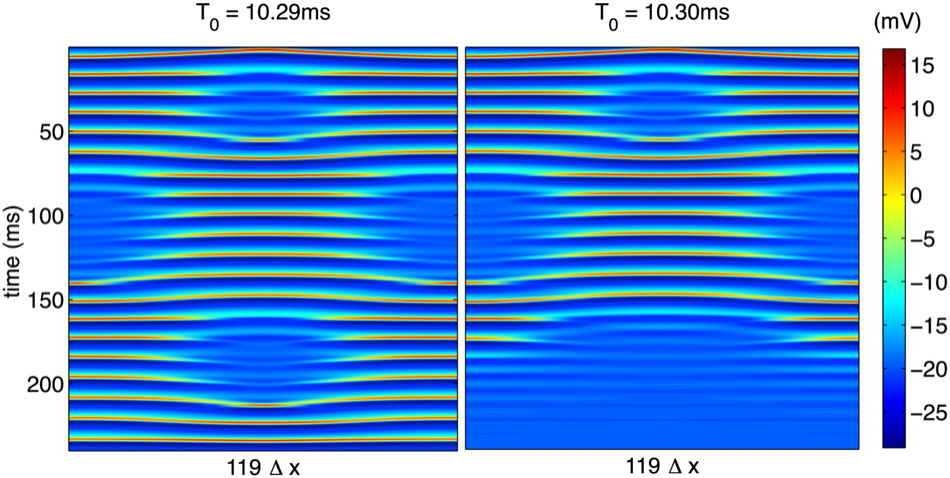
Complex spatiotemporal pattern generated with increased stimulation time near the crossover between quiescent and synchronized steady-states. A fourth-order, five-point stencil was used for evaluation of the spatial derivative. Compare to Fig. 11.

For numerical solutions of Equations (37)–(38), we discretize space and time by taking mesh sizes of Δ*x* = 0.1cm and Δ*t* = 0.01ms for spatial mesh and time-integration step size, respectively. Time integration is performed using an explicit, fixed step-size, fourth-order Runge Kutta method [40]. The spatial derivative is approximated by the standard second-order stencil,

$$\begin{array}{c} \frac{\partial^{2}V}{\partial x^{2}}\to \frac{1}{\Delta x^{2}}\left(u_{j+1}+u_{j-1}-2u_{j}\right), \end{array}$$ (42)

with a fourth-order, five-point stencil [41] used to confirm all results displayed in this section. When using explicit integration schemes, the numerical stability of a linear diffusive system is governed by the Courant-Friedrichs-Levy (CFL) number,

$$\begin{array}{c} \mu =\frac{D\Delta t}{\Delta x^{2}}, \end{array}$$ (43)

which should satisfy *μ* ≪ 1 to ensure a numerically stable solution. We find experimentally that even for *μ* ≪ 1, the solution is extremely sensitive to changes in step sizes due to the nonlinear nature of the system which precludes a strict application of linear stability analysis. To check the accuracy of solutions presented in this paper, all solutions were computed with a variety of spatial and temporal step sizes, Δ*t* and Δ*x*, respectively, while holding cable length *L* and overall integration time *T* fixed. Additionally, a fourth-order, five-point stencil was employed for the diffusive term in Equation (37) and compared to results obtained from a standard second-order, three-point stencil. Aside from Figs. 11–13, no difference was observed when varying step sizes or spatial stencil. In the transitional regime, however, simply changing the spatial stencil resulted in significantly different behavior. Qualitatively similar behavior to that shown in Fig. 11 exists when the modified stencil is employed, but the particular value of *T*_0_ at which it occurs is slightly different when the higher-order stencil is used, as shown in Fig. 13.

Regardless of the particulars of the numerical parameters used to obtain approximate solutions, the behavior depicted in Fig. 11 is robust in the sense that it can generated for some particular form of the stimulation protocol. While such delicate behavior is extremely difficult to investigate rigorously within the context of a highly nonlinear, continuous reaction-diffusion system, these results have immediate relevance to inherently discrete systems where the diffusive coupling

$$\begin{array}{ccc} \Delta tD\frac{\partial^{2}u}{\partial x^{2}} & \to & \frac{\Delta tD}{\Delta x^{2}}\left[u_{j+1}+u_{j-1}-2u_{j}\right], \end{array}$$ (44)

$$\begin{array}{ccc} & \equiv & \Gamma \left(u_{j+1}+u_{j-1}-2u_{j}\right), \end{array}$$ (45)

becomes a synaptic coupling between discrete neurons with a well-defined value. In this context, the numerical instability we see corresponds to a dramatic sensitivity of system behavior on the particular coupling between neighboring cells.

## Discussion

In this paper, we have presented a physically-motived modification to excitable models based on the charge depletion which occurs in brain neurons. By incorporating this modification into the conventional Morris-Lecar reaction-diffusion model, we have explored several examples of the emergent complex behavior which have particular relevance to synchronization of neurons in the brain and the relevance of synchronization to diseases such as epilepsy. The addition of a variable Nernst potential creates a region in parameter space in which self-sustaining oscillations, or stable limit cycles, exist without requiring external driving or leakage currents. Typical points on the boundary of this region correspond to Hopf bifurcations, and the neighborhoods of these bifurcation points support the coexistence of stable equilibria as well as stable and unstable limit cycles. The results presented in this paper are largely due to the interplay between these vastly different types of solutions.

Near the subcritical Hopf bifurcation boundary, an excitable cell firing on its stable limit cycle is susceptible to transitioning to a stable equilibrium through the application of a carefully timed stimulation current. We have demonstrated the existence of such a window of vulnerability with respect to the instantaneous phase of the system at the time the stimulation is applied. The fraction of phase occupied by this window is a tremendously complicated function of system parameters, but as depicted in Fig. 6, it may generally be expected to depend predictably on the system’s distance in (*α*, *V*_0_) space from the subcritical Hopf bifurcation, with closer points having larger windows.

By probing this vulnerability in a one-dimensional cable, we have demonstrated that the spatial extent of the system plays a critical role in the spatiotemporal dynamics generated by applying a localized stimulus to a fully synchronized system. In particular, holding all parameters fixed, we find a range of system sizes for which a localized stimulation totally disrupts the synchronized oscillations, sending the entire cable to a quiescent state. In the continuum limit, this change in system size *L* is equivalent to scaling the diffusion constant *D* while holding *L* fixed. By observing the proper scaling, we conclude that our results obtained from a finite-differencing scheme adequately model the continuum system. Furthermore, the window of vulnerability with respect to the system’s phase when the stimulus is applied may be reconstructed for the entire cable as shown in Fig. 10. The window of vulnerability for the extended cable shows a qualitatively similar picture to that of the single cell (c.f. Fig. 6) with a range of phases for which a stimulation causes the entire cable to evolve toward a homogeneous, quiescent state with no oscillations. Interestingly, the edges of this window correspond to dramatic instabilities in the one-dimensional cable where highly complex spatiotemportal patterns emerge as long-lived transient effects. The results presented in this paper serve to sketch the basic building blocks for spatiotemporal patterns which naturally emerge from this system. Clearly, detailed investigations of the possible patterns and their relation to neurophysiology are lines of direction for future work. The appearance of recent studies [42] using multiple-lead sensors to measure neuronal activity at several locations suggest our model could provide an important component in explaining measurements or predicting outcomes of future experiments.

Generalizing these reaction-diffusion models to include dynamical Nernst potentials provides a platform for future investigation into what diffusive chemical influences may have on neural network dynamics. Seizure activity and various bursting events are heavily influenced by external factors. If preliminary information regarding medicinal reactions are known, these effects could possibly be incorporated within a reaction-diffusion system by means of profiled diffusion. Recent research into Belousov-Zhabotinsky reactions and chemical computing [43] show a promising future for understanding how information is retrieved and written within networks such as these. In addition to providing benefits to those suffering from mental illness, understanding neural network stability could also provide new ways of encrypting sensitive network information.

Research on epilepsy and seizures has shown that a neuron will swell as a result of electrical activity and when modeling such activity one must account for variations in ion concentration [8, 9]. By incorporating this dynamic size variation into network simulations it was shown that if glial cells fail to maintain the proper micro-environmental conditions neurons will produce seizure-like activity. It was also suggested that how persistent states respond to perturbations may be critical to transient behavior such as working memory [19]. One could imagine a better understanding of neural networks will allow for a more quantitative understanding of complex brain activity such as working memory.

In the work of Mornev and Aslanidi [2, 7], soliton-like behavior, with reflection from zero-flux boundaries, was observed in addition to complete synchronization of the cable. The ratio of time constants for membrane potential and recovery was shifted by a function of trans-membrane potential but without any obvious physiological reason. The present work is an extension of this type of investigation with the aim of better understanding the particular nonlinearities responsible for observable phenomena and strengthening the link between mathematics and physiology. In agreement with Aslanidi and Mornev we observed elevated ion fluxes in proximity to the ion impermeable, no-flux, boundaries. Therefore, one would expect, for fibers of total size comparable to the size of the stimulation region, these no-flux boundaries would reinforce the current applied at the site of stimulation. This is in agreement with what we observed for smaller cables where the entire fiber remains synchronized with a simple phase-shift in accordance with the stimulus applied. That is, if the size of the stimulation site is relatively large in comparison to the fiber length, the no-flux boundaries will work to amplify the applied stimulus so that the fiber becomes indistinguishable from the situation where one would apply a stimulus to the entire fiber itself.

However, when the fiber is long enough to produce effective causal separation between the center and edges, yet short enough so that the interplay between the center stimulus and elevated ion-fluxes at both ends of the fiber may produce long-lived, transient behavior, we find an alternation between establishing a phase difference and complete quiescence. Interestingly, this is also the regime where pattern formation was observed. The open boundaries act as impermeable membranes so that the fiber becomes isolated, and acts as a one-dimensional pathway for voltage disturbances. If a cable is composed of cells tuned to be near a Hopf Bifurcation point, it is possible for the elevated ion-concentrations located at the fiber’s edge to cause reflections at the open boundaries [31]. Therefore, these isolated regions may sustain activity without external influence. When a stimulus is applied to these isolated regions, as would be the case for sensory information being delivered to information processing units within the brain, the transients may form complicated patterns. Most interestingly, when a single cell of the fiber is monitored we observe potential curves (c.f., Fig. 12) qualitatively identical to bursting measurements made in rat brains [44]. We do note that different classes of neurons have different bursting behaviors and waveforms, and detailed investigations of mechanisms for particular classes do exist [45]. One important goal of this paper is to demonstrate a new mechanism by which single- or few-cell bursting *may* emerge. Specifically, we demonstrate how a small, localized region within excitable tissue may exhibit bursting through the transient spatiotemporal complexity occurring within the system.

The results in this paper suggest soliton-like regimes are likely common in biological, excitable media. Additionally, we find that one need not abandon the Hodgkin-Huxley-like, parallel channel framework to observe such regimes. Examining the phase-response for single cells close to a Hopf bifurcation point, one finds the existence of a stable limit-cycle, unstable limit-cycle, and a stable equilibrium point. If the initial conditions are such that the cell is undergoing stable limit-cycle oscillations and a stimulus is applied within the hyperpolarization stage of cell recovery so that the trajectory moves across the unstable limit-cycle, then the stimulation will result in a quiescent cell. Based on these observations the notion of a “vulnerable window” was established. This compact picture of a vulnerability window represents a sort of building block to which further research may add in the construction of reaction-diffusion systems used to describe memory formation and both physiological and pathophysiological behavior within such systems.
